# Abortion after Rape

**Published:** 1915-02

**Authors:** 


**Abortion After Rape.** Von Olshausen of Berlin, quoted by the Critic & Guide, discusses the ethics of this question, which has been brought into prominence by the war. He concludes that abortion is not justified unless on account of threatened insanity of the pregnant woman.

